# Impacts of clustering on noise-induced spiking regularity in the excitatory neuronal networks of subnetworks

**DOI:** 10.3389/fncom.2015.00085

**Published:** 2015-07-07

**Authors:** Huiyan Li, Xiaojuan Sun, Jinghua Xiao

**Affiliations:** School of Science, Beijing University of Posts and TelecommunicationsBeijing, China

**Keywords:** neuronal network of subnetworks, spiking regularity, clustering, noise, excitatory neurons

## Abstract

In this paper, we investigate how clustering factors influent spiking regularity of the neuronal network of subnetworks. In order to do so, we fix the averaged coupling probability and the averaged coupling strength, and take the cluster number *M*, the ratio of intra-connection probability and inter-connection probability *R*, the ratio of intra-coupling strength and inter-coupling strength *S* as controlled parameters. With the obtained simulation results, we find that spiking regularity of the neuronal networks has little variations with changing of *R* and *S* when *M* is fixed. However, cluster number *M* could reduce the spiking regularity to low level when the uniform neuronal network's spiking regularity is at high level. Combined the obtained results, we can see that clustering factors have little influences on the spiking regularity when the entire energy is fixed, which could be controlled by the averaged coupling strength and the averaged connection probability.

## Introduction

Noise exists everywhere in biological systems. In genetic networks, random fluctuations are inevitable as chemical reactions are probabilistic and many genes, RNAs and proteins are present in low numbers per cell (Paulsson, 2004). Noise in genetic networks is multi-functional. It enables coordination of gene expression across large regulons and facilitates evolutionary transitions (Eldar and Elowitz, 2010). Meanwhile, it can also induce switches between distinct gene-expression states (Xu et al., 2013a,b).

In neuronal networks, random fluctuations are also inevitable as random exocytosis of vesicles containing neurotransmitters and random transmissions of ions inside and outside of the neuron. As in genetic networks, noise also has great influences on neuronal systems (Lindner et al., 2004). Noise can assist neurons in the detection of weak signals via a mechanism known as stochastic resonance (Stacey and Durand, 2000, 2001), and induce synchronization in neuronal networks (Zhou and Kurths, 2003; Wu et al., 2005). Except for these effects, noise also has great influences on neuronal oscillatory activities (Doiron et al., 2004; Dorval and White, 2005), neuronal information coding (Averbeck and Lee, 2006) and other neuronal dynamical behaviors (Sun et al., 2008a,b, 2010, 2011a; Ma et al., 2012; Gu et al., 2013).

In neuronal systems, neuronal information is carried by the spike trains. Firing rate and spiking times are two important statistical quantities in the transmission process of neuronal information. Spiking regularity characterizes regularity of interspike intervals of the spike trains, which has close relationship with spiking times. The more regular the interspike intervals are, the more higher the spiking regularity is. In the past, spiking regularity has been discussed in many literatures. Many factors, such as noise, time delay, and synaptic couplings, are found to have significant influences on the spiking regularity of neuronal systems. For example, it has been found that sodium channel noise enhances spiking regularity of neuronal systems, while potassium channel noise decreases it (Schmid et al., 2004a,b; Ozer et al., 2009; Sun et al., 2011b; Sun and Shi, 2014). Time delay can optimize the neuronal network's spiking regularity (Yang et al., 2012). And synaptic coupling also has great influences on it, it is found that some optimal frequencies of the time-periodic intercoupling strength could make the spiking regularity at high level (Sun and Zheng, 2014).

In previous numerical studies, spiking regularity is investigated mostly in single neuronal networks, such as regular, small-world, and scale-free networks. However, our brain cortex is a complex network. It is found to be hierarchy and clustered (or modular) (Sporns et al., 2004; Bullmore and Sporns, 2009). Currently, some works reported that clustering could play important roles in neuronal systems. For example, it has been found to negatively correlate with the performance of associative memory (Chen et al., 2011) and enhance the robustness of self-organized criticality in neuronal networks (Wang and Zhou, 2012). In recent years, synchronization, stochastic resonance and spiking regularity of clustered neuronal networks has been discussed in a few literatures (Sun et al., 2011b; Yu et al., 2011; Batista et al., 2012a,b). However, studies about influences of clustering on neuronal dynamics are less. Considering the important roles of clustering in neuronal systems, effects of clustering on neuronal dynamics of clustered neuronal networks should be discussed. Here, we pay attention to discuss effects of clustering on spiking regularity of clustered neuronal networks.

In the present paper, we will focus on discussing effects of clustering, measured by *R*, *S* (which are illustrated in the coming contents) and the cluster number *M* on spiking regularity in neuronal network of subnetworks (clustered neuronal networks). We use computer simulations to address this subject in the following contents. In the coming section, network structure is illustrated first, and then mathematical models of the discussed clustered neuronal network are presented, at last we introduce a measure to quantify the neuronal networks' spiking regularity. In the Section Results, we investigate effects of clustering on the spiking regularity in details. Finally, Discussions and Summary of the paper are presented.

## Materials and methods

### Clustered structure of neuronal networks

**Figure 1:**
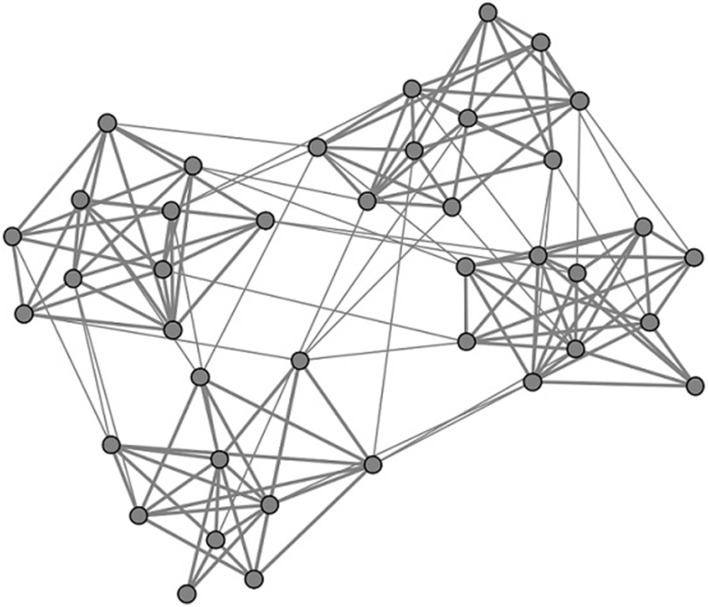
**Schematic presentation of the considered network architecture**. The whole network consists of *M* = 4 subnetworks, each containing 10 neurons. Neurons inside each subnetwork connected with each other with probability *p*_*in*_ and neurons from different subnetworks connected with each with probability *p*_*out*_. Here the ratio *R* = *p*_*in*_∕*p*_*out*_ is taken as 15 and the connections probability between all neuron inside the whole network is taken as 0.15.

In this paper, neuronal network is considered to have clustered structure. It totally contains *N* excitatory neurons, which are divided into *M* subnetworks equally. Namely, there are *N*∕*M* neurons in each subnetwork. Here we set *N* = 200. Neurons inside the network are linked randomly. Neuron pairs in the same subnetwork are linked with the probability *p*_*in*_, while neuron pairs from different subnetworks are linked with the probability *p*_*out*_. Higher values of *R* = *p*_*in*_∕*p*_*out*_ favor connections within a local subnetwork over non-local connections. In this paper, the quantities *p*_*in*_ and *p*_*out*_ are chosen so that the connection probability between excitatory neurons remained 0.05 when averaged across all pairs (Kumar-Litwin and Doiron, 2012). *p*_*in*_ and *p*_*out*_ are named as intra-connection probability and inter-connection probability here, respectively. In Figure 1, an example of the considered network topology is shown. In this figure, which serves illustrative purposes, there are four subnetworks, each consisting of 10 neurons. Neurons inside each subnetwork connected with each other with the probability *p*_*in*_ and neurons from different subnetworks connected with each with the probability *p*_*out*_. Here the ratio *R* = *p*_*in*_∕*p*_*out*_ is taken as 15 and the connections probability between all neuron inside the whole network is taken as 0.15. In Figure 1, the connections inside subnetworks are thicker than the connections between different subnetworks. This indicates that the coupling strength of neurons inside subnetworks is larger than the coupling strength of neurons between different subnetworks, which will be illustrated in details in the following contents.

### Mathematical models of the clustered neuronal network

The FitzHugh–Nagumo(FHN) model (Fitzhugh, 1961; Nagumo et al., 1962) is used to describe the local single neuron, and the equations of the clustered neuronal network are present as followings:

$$\{\begin{array}{c} \varepsilon {\dot{x}}_{i}=x_{i}-\frac{x_{i}^{3}}{3}-y_{i}+\sum_{j=1}^{N}J_{ij}g_{ij}\left(x_{j}-x_{i}\right)\\ {\dot{y}}_{i}=x_{i}+a_{i}+D\xi_{i}\left(t\right) \end{array}$$

where *x* represents the action potential and is a fast variable, *y* represents the slow recovery variable. The subscript *i* represent the *i*-th neuron inside the clustered network. The time separation between fast and slow variable is determined by the small quantity ε, which is set as 0.01. The matrix *J* = (*J*_*ij*_) is a connection matrix of the neuronal network: *J*_*ij*_ = 1 if neuron *i* connects to neuron *j*; *J*_*ij*_ = 0 otherwise.

*g*_*ij*_ is the coupling strength between the *i*-th neuron and the *j*-th neuron. *g*_*ij*_ is set to be *g*_*in*_ if the *i*-th neuron and *j*-th neuron are in the same subnetwork, otherwise it is set to be *g*_*out*_. The ratio *S* = *g*_*in*_∕*g*_*out*_ is taken as another clustering measure. The quantities *g*_*in*_ and *g*_*out*_ are chosen so that the averaged coupling strength between excitatory neurons is kept as *g*, value of *g* is given in the following contents. ξ_*i*_(*t*) is Gaussian noise with $\langle \xi_{i}\left(t\right)\rangle =0$ and $\langle \xi_{i}\left(t\right)\xi_{j}\left(t^{\text{`}}\right)\rangle =\delta_{ij}\delta \left(t-t^{\text{`}}\right)$ (Fox, 1989; Li et al., 2012). And *D* is the noise intensity of ξ_*i*_(*t*).

For a single FHN neuron without any external stimulus, it is at an excitable state if *a*_*i*_ > 1. In this paper, we let all *a*_*i*_ being larger than 1.0 and uniformly distributed in the interval (1.0, 1.1) (*a*_*i*_ ~ *U* (1.0, 1.1)). Namely, all neurons inside the networks are at their steady states and do not generate any firing pulses in the absence of external noise ξ_*i*_(*t*).

Stochastic Euler algorithm is applied in the simulation process with time step being 0.001 and the Gaussian noise ξ_*i*_(*t*) is generated by using Box–Mueller algorithm (Knuth, 1969; Fox, 1989). The initial values are set as *x*_*i*_(0) = 0, *y*_*i*_(0) = 0. In the followings, we will take the parameters *R*, *S*, *M* as controlled parameters to study effects of clustering on spiking regularity of the excitatory neuronal network of subnetworks. And the clustering measures *R*, *S* are always larger than 1. All the results are averaged by 10 realizations.

### Measure of spiking regularity

In order to quantitatively characterize spiking regularity of the neuronal networks, we introduce a measure λ, which is defined as

$$\lambda =\frac{1}{N}\sum_{i=1}^{N}\lambda_{i}$$

Here, *N* is the total number of neurons in the neuronal network. λ_*i*_ is the reciprocal of the coefficient of variation and it can quantify the regularity of spike timing in a neuron (Gerstner et al., 2014). λ_*i*_ is formulated as

$$\lambda_{i}=\frac{\langle T_{i,k}\rangle }{\sqrt{\langle T_{i,k}{}^{2}\rangle -{\langle T_{i,k}\rangle }^{2}}}$$

where *T*_*i*,*k*_ = *t*_*i*,*k*+1_ − *t*_*i*,*k*_ represents the inter-spike interval with *t*_*i*,*k*_ denoting the time of the *k*-th spike of the *i*-th neuron; 〈*T_i,k_*〉 and 〈*T_i,k_*^2^〉 denote the mean and the mean squared inter-spike intervals, respectively. Spiking times are defined by the upward crossing of the membrane potential *V* past a certain value *V*_*th*_ (here *V*_*th*_ is taken as 0 mV). Note that the threshold value can vary in a wide range without altering the results. Larger λ indicates higher spiking regularity of the entire neuronal network.

## Results

Before discussing effects of clustering parameters *S*, *R*, and *M* on spiking regularity, we first study how coupling strength affects it in an uniform neuronal network. Here the uniform neuronal network indicates the neuronal network without clusters. With the aid of results about dependence of spiking regularity on the coupling strength *g*, we could have a deep understanding of effects of clusterings on the neuronal network's spiking regularity.

### Dependence of spiking regularity on the averaged coupling strength *G* in uniform neuronal networks

**Figure 2:**
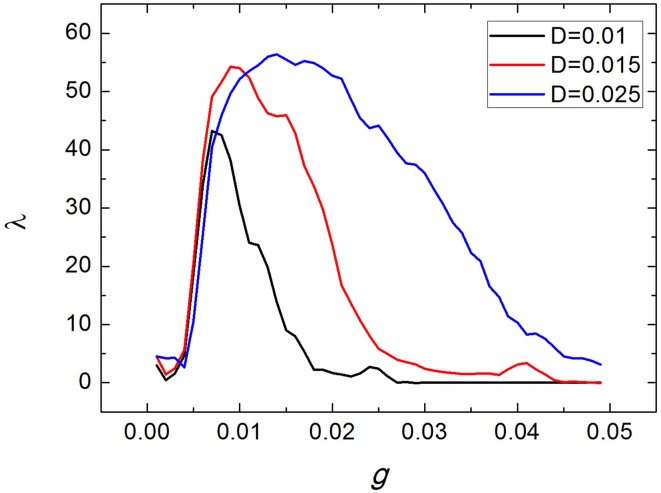
**Dependence of spiking regularity of the uniform neuronal networks on the coupling strength** ***g*** **for various values of noise intensity** ***D***.

Dependence of spiking regularity of the uniform neuronal networks on the averaged coupling strength *g* for various values of noise intensity *D* are exhibited in Figure 2. From Figure 2, we can see that for each *D*, there exist some optimal values of *g* at which the uniform neuronal networks generate neuronal firing activities with higher spiking regularity. For example, when *D* = 0.015, with the averaged coupling strength *g* increasing, λ increases first and then decreases, with a maximum of λ appears at about *g* = 0.01.

**Figure 3:**
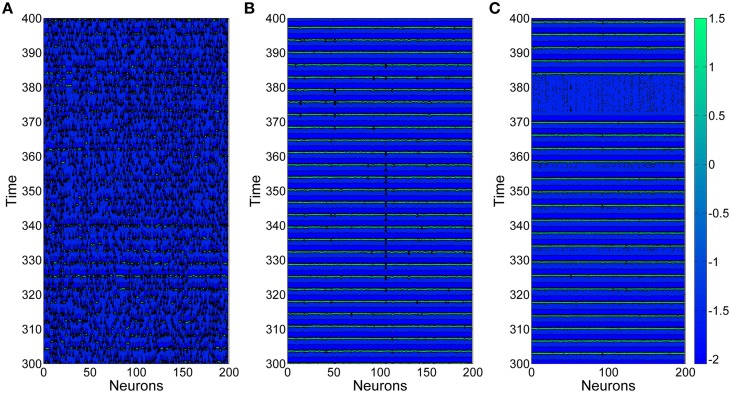
**Spatiotemporal patterns of the uniform neuronal networks for different coupling strength** ***g***. **(A)** *g* = 0.004; **(B)** *g* = 0.01; **(C)** *g* = 0.025. Here *D* = 0.015.

Figure 3 shows three typical spatiotemporal patterns observed in the neuronal network for different values of the coupling strength *g*, here *D* = 0.015. Obviously, the coupling strength can give great influences on firing behaviors of neuronal networks. When the coupling strength *g* is small, the spatiotemporal firing patterns are turbulent (or disordered), as illustrated in Figure 3A. This is because neurons generate firings almost by their own way when the coupling strengths between them are weak. However, when the coupling strength *g* = 0.01, as shown in Figure 3B, neurons generate spikes synchronously and the interspike intervals are nearly equivalent. If we further increase coupling strength *g* (for example *g* = 0.025), neurons inside the neuronal network still keep synchronous, but the interspike intervals become irregular, which leads spiking regularity being lower, as exhibited in Figure 3C.

In the followings, we take *D* = 0.015. And in order to investigate effects of clusterings on the spiking regularity, we set the coupling strength *g* as 0.004, 0.01, 0.025, respectively. Namely, we will investigate whether clustering factors have different effects when the neuronal networks are at different spiking regularity states, as shown in Figure 3.

### Effects of clustering measure *M* on the spiking regularity

**Figure 4:**
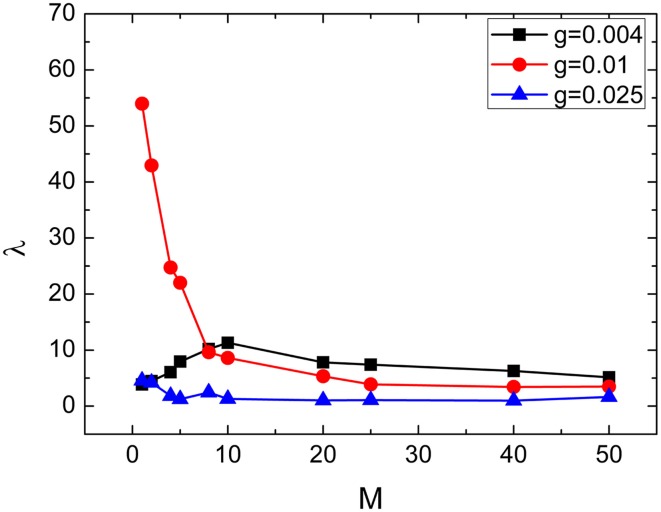
**Dependence of spiking regularity λ on the cluster number** ***M*** **for three different values of coupling strength** ***g***. Here *D* = 0.015, *R* = 20, and *S* = 30.

**Figure 5:**
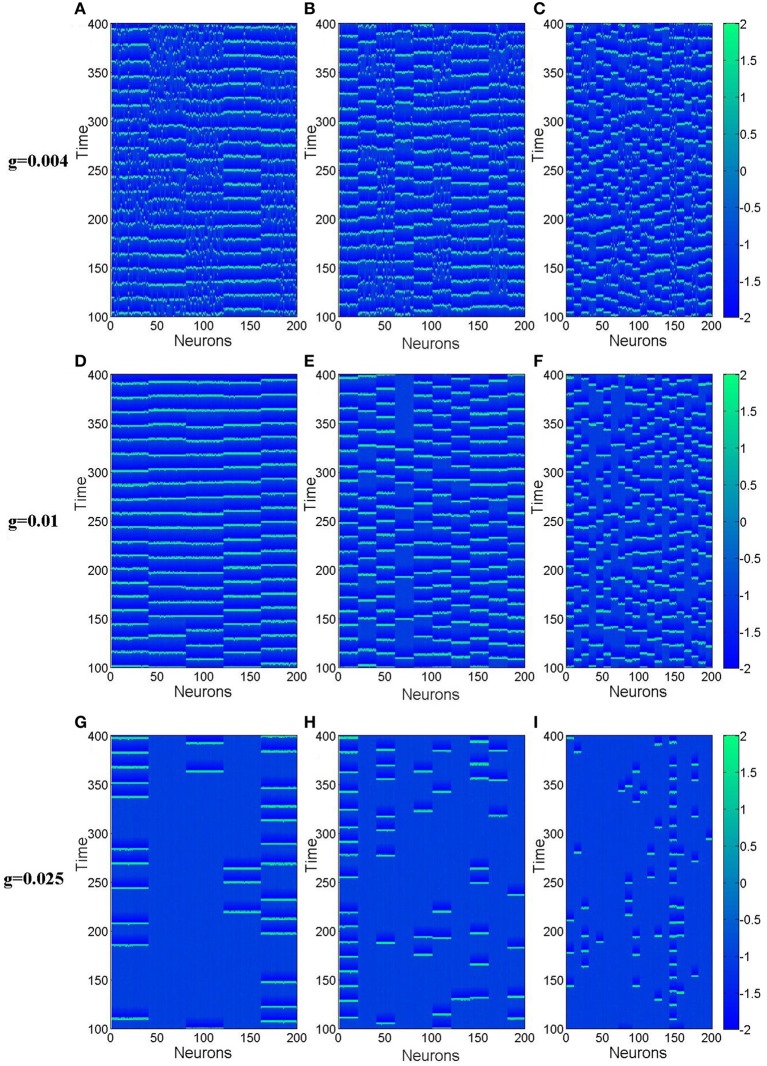
**Spatiotemporal patterns of the clustered neuronal networks for different cluster number** ***M***. **(A–C)** *g* = 0.004; **(D–F)** *g* = 0.01; **(G–I)** *g* = 0.025. Here *D* = 0.015, *R* = 20, and *S* = 30.

From this subsection, we begin to discuss effects of clusterings on the neuronal networks' spiking regularity. Firstly, we investigate how the cluster number *M* influents it. Here, the ratio *R* = *p*_*in*_∕*p*_*out*_ is set as 20 and the ratio *S* = *g*_*in*_∕*g*_*out*_ is set as 30. Figure 4 exhibits dependence of the spiking regularity λ on cluster number *M* for three different coupling strength *g*. As discussed in the above subsection, the uniform neuronal networks' spiking regularity is lower when *g* = 0.004 and 0.025, while they could take almost highest spiking regularity when the coupling strength *g* is optimal, for example *g* = 0.01 when the noise intensity *D* = 0.015. From Figure 4, we can see that when the neuronal network has clustered structures, the spiking regularity measured by λ decreases with *M* for *g* = 0.01, and it does not change too much for *g* = 0.004 and 0.025. In order to observe the dependence of spiking regularity on *M* clearly, nine spatiotemporal patterns for three different values of *M* with *g* = 0.004, 0.01, and 0.025 are presented in the Figure 5, respectively. In the Figure 5, *M* is taken as 5 for Figures 5A,D,G, 10 for Figures 5B,E,H, and 20 for Figures 5C,F,I. For *g* = 0.004 and 0.025, the interspike intervals of each neuron observed from the corresponding spatiotemporal patterns shown by the Figures 5A–C,G–I are always irregular no matter what value of *M* takes. It indicates the clustered neuronal networks' spiking regularity always stay at lower levels, i.e., *M* has little influences on the spiking regularity in these cases. For *g* = 0.01, with increasing of clustering number *M*, the interspike intervals of each neuron change from regular to irregular as exhibited by Figures 3B, 5D–F, which indicates decreasing of spiking regularity of the clustered neuronal network. With these obtained results, we can see that the cluster number *M* could have some influences on the spiking regularity just when the uniform neuronal networks are at high levels (rf. *g* = 0.01). Furthermore, compared the spatiotemporal patterns exhibited in Figure 3 with the corresponding ones shown in Figure 5, we can see that the spatiotemporal patterns are split into more and more stripes with increasing of clustering number *M*. Here, we take Figures 3B, 5D–F with *g* = 0.01 as examples. Compared with Figure 3, we can clearly see five strips in Figure 5D where *M* = 5. And with *M* increases further to 10 and 20, the strips in the spatiotemporal patterns increase to 10 and 20 correspondingly, as shown in Figures 5E,F. In fact, every strip region indicates one cluster. Because inter-coupling strength is large enough and larger than intra-coupling strength, neurons inside each cluster are synchronized with each other but not synchronized with other neurons from other clusters. This is why we can clearly observe strips in Figures 5D–F.

With the introduction of clusters, the ratio of the intra-connection probability *p*_*in*_ to the inter-connection probability *p*_*out*_ and the ratio of the intra-coupling strength *g*_*in*_ to the inter-coupling strength *g*_*out*_ are also introduced. Therefore, we may wonder how these two clustering factors affect the spiking regularity? In order to give answer to this question, we will focus on discussing effects of *R*, *S* on λ in details in the following subsection.

### Effects of clustering measures *S* and *R* on the spiking regularity

**Figure 6:**
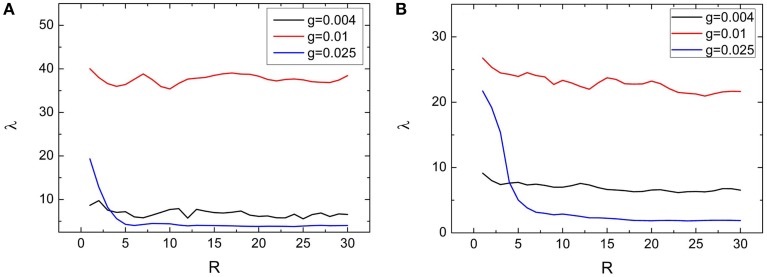
**Dependence of spiking regularity λ on the ratio** ***R*** **for three different values of coupling strength** ***g*** **with** ***M*** **= 2 (A) and** ***M*** **= 4 (B)**. Here *D* = 0.015, *S* = 30. (colored on line).

**Figure 7:**
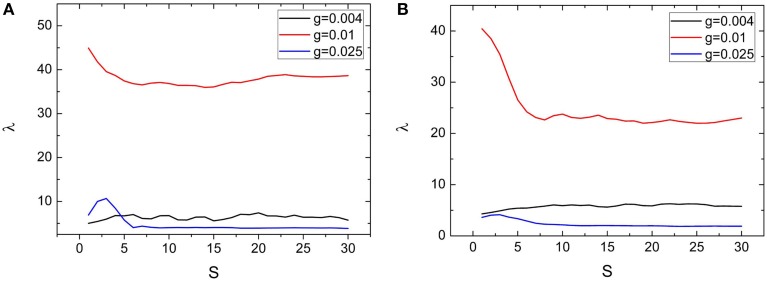
**Dependence of spiking regularity λ on the ratio** ***S*** **for three different values of coupling strength** ***g*** **with** ***M*** **= 2 (A) and** ***M*** **= 4 (B)**. Here *D* = 0.015, *R* = 20. (colored on line).

Dependences of spiking regularity on the clustering factors *R* and *S* are presented by Figures 6, 7, respectively. In the Figures 6A, 7A, variations of λ on *R* and *S* for *M* = 2 are exhibited. From these two figures, we can see that λ just has small fluctuations when *R* and *S* changes for *g* = 0.004 and 0.01. When *M* = 2, for *g* = 0.025, combined with other clustering factors, the spiking regularity decreases with *R* quickly and then stays at a low level, as shown in Figure 6A; And the spiking regularity increases a little with *S* at first and then decreases to a low level, as shown in Figure 7A. When *M* = 4, we observe similar phenomena as *M* = 2, detailed simulation results are shown in Figures 6B, 7B. From these results, we can see that the clustering factors *R* and *S* has little influences on the spiking regularity. It means that for the current clustered neuronal networks, the averaged coupling strength *g* and the averaged connection probability *p* play the dominant role on controlling the spiking regularity. When we keep these two parameters being constants and just change the allocations between intra and inter connections, the spiking regularity of the whole clustered neuronal networks does not vary too much and is almost determined by values of parameters *g* and *p*.

## Discussions and summary

In neuronal systems, synchronous firings of neurons is another important non-linear phenomenon and has been discussed in many literatures in the past. Interacting neurons may exhibit different forms of synchrony, such as complete synchronization, phase synchronization of spikes and bursts (Hu and Zhou, 2000; Vreeswijk and Hansel, 2001; Dhamala et al., 2004; Belykh et al., 2005; Wang et al., 2009). For complete synchronization, standard deviation of membrane potential of neurons is usually used to quantify a neuronal system's synchrony (Wang et al., 2009). While for phase synchronization, order parameter is the mostly used measure (Hu and Zhou, 2000; Vreeswijk and Hansel, 2001; Dhamala et al., 2004; Belykh et al., 2005). In this paper, we discuss about spiking regularity quantified by the reciprocal of the coefficient of variation of interspike intervals. The measure we used here can quantify the width of the interval distribution, then in turn can quantify how regular of the interspike intervals. Thus, the measure we used here is different from the other measures which is used to quantify synchronous firing activities. In fact, from the obtained results we can also see the difference between spiking regularity and synchronous firings. As shown in Figures 3B,C, neuronal network exhibits synchronous firings but spiking regularity of these two spatiotemporal patterns is different, one has high spiking regularity (Figure 3B) and the other's spiking regularity is low (Figure 3C). Namely, lower spiking regularity of a neuronal network does not indicate less synchrony; And higher synchronous firings also does not imply higher spiking regularity. This relationship between synchronous firings and spiking regularity of neuronal network has also been reported in other works (Hu and Zhou, 2000).

In this paper, we investigate how clustering factors influent spiking regularity of neuronal networks. FitzHugh–Nagumo models are used as building blocks here. In order to investigate the clustering effects, we fix the averaged coupling probability *p* and the averaged coupling strength *g*. Then we take the cluster number *M*, the ratio of intra-connection probability and inter-connection probability *R*, the ratio of intra-coupling strength and inter-coupling strength *S* as controlled parameters to discuss effects of clusterings on the spiking regularity.

In order to have a deep understanding on the clustering effects, we first presented dependence of the spiking regularity of an uniform neuronal network (i.e., there is no clusters inside the neuronal network) on the averaged coupling strength *g* for different values of noise intensity *D*. From these results, we found that the uniform neuronal network exhibited three typical spatiotemporal patterns, two of them with low spiking regularity. We started from these three spatiotemporal patterns to discuss how clusterings influent spiking regularity of the clustered neuronal network. By introducing clusters inside the neuronal network, we found that the cluster number *M* could have great influences on the spiking regularity when the uniform neuronal network's spiking regularity stays at high level. While when we fix the cluster number *M* and varies the two ratios *R* and *S* (note that the average coupling strength *g* and the averaged connection probability *p* are fixed), we found that these two parameters has little influences on the spiking regularity. Combined with the obtained results, we found that spiking regularity of the clustered neuronal networks is almost controlled by the averaged coupling strength *g* and the averaged connection probability *p*.

### Conflict of interest statement

The authors declare that the research was conducted in the absence of any commercial or financial relationships that could be construed as a potential conflict of interest.
